# Articles That Use Artificial Intelligence for Ultrasound: A Reader’s Guide

**DOI:** 10.3389/fonc.2021.631813

**Published:** 2021-06-10

**Authors:** Ming Kuang, Hang-Tong Hu, Wei Li, Shu-Ling Chen, Xiao-Zhou Lu

**Affiliations:** ^1^ Department of Medical Ultrasonics, Ultrasomics Artificial Intelligence X-Lab, Institute of Diagnostic and Interventional Ultrasound, The First Affiliated Hospital of Sun Yat-Sen University, Guangzhou, China; ^2^ Department of Hepatobiliary Surgery, The First Affiliated Hospital of Sun Yat-sen University, Guangzhou, China; ^3^ Department of Traditional Chinese Medicine, The First Affiliated Hospital of Sun Yat-sen University, Guangzhou, China

**Keywords:** ultrasound, artificial intelligence, machine learning, deep learning, radiomics

## Abstract

Artificial intelligence (AI) transforms medical images into high-throughput mineable data. Machine learning algorithms, which can be designed for modeling for lesion detection, target segmentation, disease diagnosis, and prognosis prediction, have markedly promoted precision medicine for clinical decision support. There has been a dramatic increase in the number of articles, including articles on ultrasound with AI, published in only a few years. Given the unique properties of ultrasound that differentiate it from other imaging modalities, including real-time scanning, operator-dependence, and multi-modality, readers should pay additional attention to assessing studies that rely on ultrasound AI. This review offers the readers a targeted guide covering critical points that can be used to identify strong and underpowered ultrasound AI studies.

## Introduction

By looking into pixels not readily visible to the human naked eyes, artificial intelligence (AI) has led medical imaging into the era of big data (1). Articles using conventional machine learning (ML) algorithms and deep learning, especially convolutional neural networks (CNN), have also become more numerous over the past several years. Studies have reported the use of AI in X-rays, computerized tomography (CT), magnetic resonance imaging (MRI), ultrasound, and other types of scans, and they have reported superior performance of AI to that of conventional methods in disease detection, characterization, and patient prognosis prediction (2–4).

Working groups of the Consolidated Standards of Reporting Trials-Artificial Intelligence (CONSORT-AI) and the Standard Protocol Items: Recommendations for Interventional Trials-Artificial Intelligence (SPIRIT-AI) have developed an extension to the core CONSORT 2010 items and 2013 SPIRIT statement that serves as a guidance for medical AI studies (5, 6). Given the rapid expansion of the literature published, *JAMA* has provided a reader’s guide to assessing clinical AI articles (7), which reviewed the basics of machine learning and aspects of the clinical implementation of AI. The editorial board of *Radiology* also highlighted several crucial considerations meant to formalize AI methodology in medical imaging studies (8). However, when AI is used with ultrasound, issues become complicated for the current existing guides.

Ultrasound uses the reflection of the ultrasonic beam to reveal tissue structure. It is one of the most widely used methods of imaging in clinical practice. It serves as a mainstay in obstetricians, cardiology, interventional therapy guidance and post-treatment surveillance (9). Ultrasound-based radiomics studies, called ultrasomics (10), follow the standard three-step AI process for medical imaging: data preparation, model development and testing, and evaluation of clinical effectiveness (11). However, given ultrasound’s unique properties of real-time scanning, operator-dependence, and multi-modality, some specific issues may influence the performance of AI models and the generalizability of a study’s results. For example, operator dependence may influence the use of expert-dataset-based model training to the resident-dataset-based model testing and use in primary hospitals. In this minireview, we aim to provide the readers with an overview of how to assess medical imaging AI articles, including some specific points regarding ultrasound AI studies.

## Objective: Is the Clinical Scenario Clearly Defined?

The objective of a medical imaging AI study should comply with two principles: first, it must be derived from clinical practical needs, and second, it must be applicable to AI technique. For example, un-enhanced ultrasound is recommended for monitoring populations at high risk of liver cancer (12), so it would be a risk stratification tool. An unenhanced ultrasound AI tool would ideally increase the detection rate of liver lesions and assist in risk assessment. When transformed into AI tasks, target recognition and classification are both technically feasible.

## Materials and Methods: Is There an Independent Testing Dataset Besides the Training and Validation Sets?

AI models are prone to overfitting. Both conventional ML and CNN algorithms can vary greatly in performance across different data sources (13). After a model is trained using the training set, its hyperparameters must be tuned in the validation set (also called the tuning set) for better generalizability. If multiple models had been trained, the validation set could also be used to select models. Once a model is finalized, its performance must be evaluated in a testing set, which has no overlap with the training or validation sets. Ideally, the testing set comes from other centers, which involves data from different ultrasound devices and vendors, and patients with different demographic characteristics. A study that reports generalizable results in an independent testing dataset would be much more valuable than a study that relies on internal validation or single-dataset-based cross-validation.

## Materials and Methods: Is the Image Processing Procedure Clearly Described?

A clear description of the image processing procedure is vital for the assessment of study repeatability and reproducibility. Readers should pay attention to the ultrasound data acquisition process and the validity of the data range. Questions below should be raised when acquiring such information. Is the data collected retrospectively or prospectively? Which modality does the study apply? Is it radio frequency signal, grayscale, elastography, doppler imaging, contrast-enhanced ultrasound (CEUS), or transferring between modalities (14, 15)? Also, the number of pictures per patient enrolled for the training or testing and whether the patients’ clinical data are involved in the AI development should be inspected.

In terms of ultrasound data preprocessing, each step should be presented clearly. Ultrasound images are derived from various devices produced by different radiologists. Ultrasound is highly operator-dependent (16, 17), which causes variations in image quality, target lesion identification, and selection of representative sections. Cropping is widely adopted in image processing in medical AI studies, and it filters out most irrelevant, non-lesion information, and for the ultrasound, reduces image heterogeneity by adjusting size and depth. Augmentation can enrich data diversity, and it can simulate the common causes of image heterogeneity as observed under real-world conditions in ultrasound examinations (18, 19). For example, resizing reduces resolution variation of different devices, rotation simulates scanning from different angles and sections, and contrast adjustment simulates variation in gain and dynamic range.

## Materials and Methods: Is the Algorithm for Modeling Suitable?

Conventional ML algorithms such as logistic regression, support vector machine (SVM), random forest, and Naïve Bayes have much fewer parameters than deep learning algorithms. For example, SVM has only 13 parameters to be adjusted, while the ResNet-50 has an amount of 2.3×10^7^ parameters. Thus, conventional ML algorithms require far less training than deep learning algorithms do (20). With a limited sample size, such as a set of only hundreds of images (not videos), conventional ML algorithms are preferred (21). However, with thousands or millions of images, deep learning algorithms, principally CNN in imaging analysis, are recommended. The minimum number of training images needed varies across different tasks and algorithms and may only be determined by evaluating the relationship between its increase and changes in model performance.

Algorithms’ clinical intelligibility, which means the level of understandability of an algorithm in a clinical way, should also be considered. There has not been any ultrasound-specific imaging analysis algorithms reported. Instead, model algorithm selection is primarily based on the type of task. Ultrasound has multiple modalities. CEUS videos record a lesion’s hemodynamic information revealed by the dynamic perfusion of microbubble contrast agents. Multi-phase image features can be extracted by simply analyzing frames from each phase but the time sequencing features were missing. Recurrent neural networks (RNNs) such as long short-term memory (LSTM) or gated recurrent units can be incorporated to these time-dimension-related tasks (18). Previous studies using LSTM in CEUS reported excellent performance (22, 23). The application of clinically explicable AI algorithms to modeling renders the study findings more clinically acceptable.

## Materials and Methods: Is the AI Algorithm Publicly Available?

Even being generalizable among different datasets in a given study, especially for studies carried out in a single center, AI performance still needs a broad verification. The existing public medical imaging data sets are minimal (24), and no public ultrasound dataset exists. Authors are encouraged to make their AI models publicly available *via* such websites as GitHub (https://github.com/) to allow independent validation, fine-tuning, and updating. A study reporting publicly available AI algorithms may improve its results’ reliability in this way.

## Results: How Do the Results Produced by the AI Model Compare to Those Produced by Expert Radiologists?

Medical AI must be evaluated against the performance of radiology experts (8). The value of a prospectively designed AI performance testing procedure can be determined by comparing its performance to that of human experts under real-world conditions. In retrospectively designed studies, missing data, and data mismatch regarding the target lesion are unavoidable in datasets collected from clinical practice, considering which is beyond AI’s ability (25). Radiologists make ultrasound diagnosis in real time during face-to-face examinations, where they receive far more information than retrospective image review does. The common study design usually underestimates radiologists’ performance and renders meaningful evaluation of medical AI difficult.

Combing clinician experience and AI’s advantages can render imaging more efficient and accurate than either alone (26). Because ultrasound offers diagnosis in real time and is heavily dependent on the operator, ultrasound AI’s performance should be compared to that of radiologists with varied experiences to develop a viable human-AI interaction strategy (27). Ideally, this strategy would involve dynamic assessment during an ultrasound examination. A specific application scenario based AI developing and testing study would have considerable practical value.

## Results: Are the Evaluation Indexes Suitable?

For detection and classification purposes, an AI model is first evaluated by the receiver operating characteristic curve (ROC) or precision-recall curve (PRC), and further by its accuracy, error rate or F1 value. However, in medical imaging analysis programs, performance is assessed based on indicators of clinical significance, such as sensitivity and specificity for diagnosis and prediction programs (28, 29), detection rate for disease screening and lesion detection (30, 31), κ and dice coefficient for inter-annotator agreement and overlapping in radiotherapy planning (32, 33). For example, for a screening task model, detection rate and sensitivity would be the primary indexes for model evaluation, while for diagnostic tasks, high specificity or positive predictive value would be the top priority. A specifically preferred high evaluation index can be achieved using an appropriate cutoff value for AI outputs but not necessarily by the default of 0.5 or the Youden index.

## Discussion: Are the Results Compared to State-of-Art Reports?

AI results should be compared to state-of-art reports, both the previous studies of the same design and these using other imaging modalities, traditional methods, or guideline recommendations. Readers should keep in mind that results without independent tests or internally validated results are not comparable to studies reporting independently tested results, no matter how good the statistics are relative to state-of-art results. A well-designed study with practical results is much more valuable than studies with flawed design but with good statistical results.

## Discussion: What Is the Unsolved Problem of the Present Work?

Limitations of medical AI studies are often the challenge of future work. For example, what situation wouldn’t the AI system be implemented when considering that AI performance errors and failure cases could influence clinical practice decision-making? What are the latent factors keeping AI systems from generalizing to other centers and populations, given the hardware requirements, algorithm versions, data quality, and processing procedures? How can these be solved in further study? Is the sample size large enough to build a robust model? The relationship between the training dataset size and model performance should be evaluated, as Dunnmon et al. (34) in the research reporting that the AI performance benefited little after a certain number of images were used for training.

## Conclusion

Given ultrasound’s unique properties, readers should pay additional attention when assessing an AI study that relies on ultrasound than those that rely on other imaging modalities. Here, we list several crucial points to help readers distinguish strong ultrasound AI articles from underpowered articles. With more formalized standards for medical AI studies published in the future, ultrasound AI studies may better benefit the clinical practice.

## Author Contributions

Conception, design, and final approval of the manuscript, MK. Preparing the main manuscript, H-TH. Editing and review of the manuscript, WL, S-LC. Revision of the manuscript, X-ZL. All authors contributed to the article and approved the submitted version.

## Funding

This work is supported by the National Natural Science Foundation of China (NO. 81971630).

## Conflict of Interest

The authors declare that the research was conducted in the absence of any commercial or financial relationships that could be construed as a potential conflict of interest.

## Supplementary Material

The Supplementary Material for this article can be found onlineat: https://www.frontiersin.org/articles/10.3389/fonc.2021.631813/full#supplementary-material
