# Supplementary material for: Articles That Use Artificial Intelligence for Ultrasound: A Reader’s Guide
Source: Front Oncol. 2021 Jun 10;11:631813. doi: 10.3389/fonc.2021.631813 (PMC8222674; doi:10.3389/fonc.2021.631813)
Supplement: Supplementary file 1 [file Table_1.docx]

Table: Key points to assess ultrasound AI articles.

| Sections | Questions | Points |
| --- | --- | --- |
| **Objective** | Is the clinical scenario clearly defined? | 1. Clinical practical needs 2. Translatable to AI tasks |
| **Materials and Methods** | Is there an independent testing dataset besides the training and validation sets? | 1. Single-dataset-based cross-validation 2. Internal validation 3. Independent test |
|  | Is the image processing procedure clearly described? | 1. Cropping 2. Augmentation |
|  | Is the algorithm for modeling suitable? | 1. Sample size 2. Clinical intelligibility |
|  | Is the AI algorithm publicly available? | GitHub |
| **Results** | How do the results produced by the AI model compare to those produced by expert radiologists? | 1. Prospective design 2. Application scenario |
|  | Are the evaluation indexes suitable? | 1. ROC, PRC, Accuracy, F1 2. SEN, SPE, PPV, NPV 3. Detection rate 4. κ and dice coefficient |
| **Discussion** | Are the results compared to state-of-art reports? | 1. Guidelines 2. Conventional methods 3. Previous studies |
|  | What is the unsolved problem of the present work? | 1. Implementable 2. Generalizable 3. Sample size estimation |

AI: artificial intelligence, ROC: Receiver Operating Characteristic Curve; PRC: precision-recall curve; SEN: Sensitivity, SPE: specificity, PPV: positive predictive value, NPV: negative predictive value.
